# Synthesis and Applications of Silver Nanowires for Transparent Conductive Films

**DOI:** 10.3390/mi10050330

**Published:** 2019-05-16

**Authors:** Yue Shi, Liang He, Qian Deng, Quanxiao Liu, Luhai Li, Wei Wang, Zhiqing Xin, Ruping Liu

**Affiliations:** 1 School of Printing and Packaging Engineering, Beijing Institute of Graphic Communication, Beijing 102600, China; shiyuematerials@gmail.com (Y.S.); dengqianpacking2@gmail.com (Q.D.); liuquanxiao@bigc.edu.cn (Q.L.); liluhai@bigc.edu.cn (L.L.); wangwei@bigc.edu.cn (W.W.); zhiqingxin@bigc.edu.cn (Z.X.); 2 State Key Laboratory of Advanced Technology for Materials Synthesis and Processing, Wuhan University of Technology, Wuhan 430070, China; hel@whut.edu.cn

**Keywords:** silver nanowires, polyol method, flexible device, transparent conductive electrodes, inkjet printing

## Abstract

Flexible transparent conductive electrodes (TCEs) are widely applied in flexible electronic devices. Among these electrodes, silver (Ag) nanowires (NWs) have gained considerable interests due to their excellent electrical and optical performances. Ag NWs with a one-dimensional nanostructure have unique characteristics from those of bulk Ag. In past 10 years, researchers have proposed various synthesis methods of Ag NWs, such as ultraviolet irradiation, template method, polyol method, etc. These methods are discussed and summarized in this review, and we conclude that the advantages of the polyol method are the most obvious. This review also provides a more comprehensive description of the polyol method for the synthesis of Ag NWs, and the synthetic factors including AgNO_3_ concentration, addition of other metal salts and polyvinyl pyrrolidone are thoroughly elaborated. Furthermore, several problems in the fabrication of Ag NWs-based TCEs and related devices are reviewed. The prospects for applications of Ag NWs-based TCE in solar cells, electroluminescence, electrochromic devices, flexible energy storage equipment, thin-film heaters and stretchable devices are discussed and summarized in detail.

## 1. Introduction

**Figure 1:**
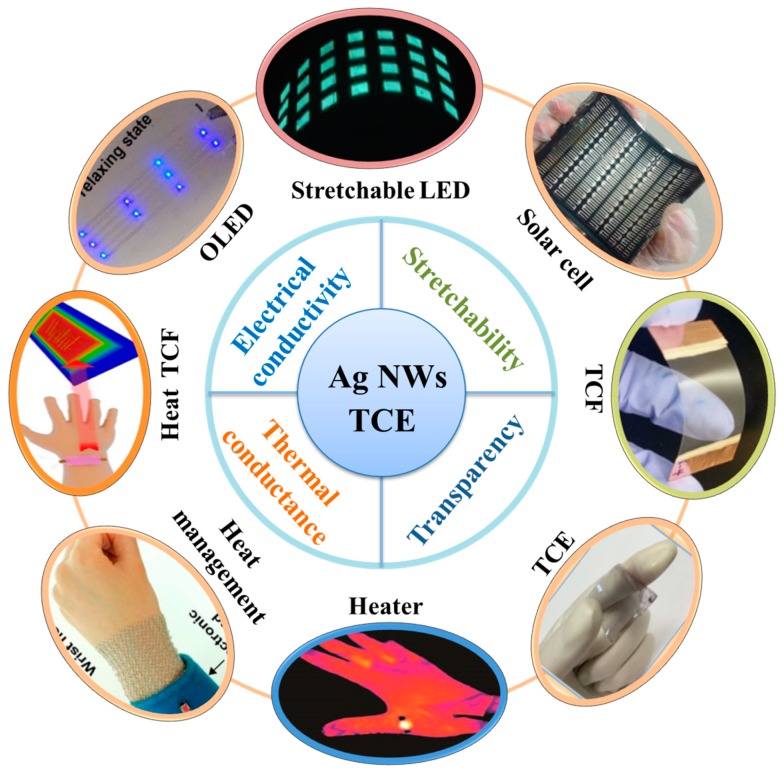
Properties and applications of recently developed devices based on Ag NWs TCE. “Heat management”, reproduced with permission [14]. Copyright 2015, ACS Nano. “Heat TCF” means “Heat Transparent conductive film”, reproduced with permission [15]. Copyright 2019, ACS Applied Materials & Interfaces. “Organic light emitting diode (OLED)”, reproduced with permission [16]. Copyright 2017, Nanotechnology. “Stretchable LED”, reproduced with permission [17]. Copyright 2017, Current Applied Physics. “Solar cell”, reproduced with permission [18]. Copyright 2016, Journal of Materials Chemistry A. “TCF”, reproduced with permission [19]. Copyright 2016, Solar Energy Materials and Solar Cells. “TCE”, reproduced with permission [20]. Copyright 2017, Solar Energy Materials and Solar Cells. “Heater”, reproduced with permission [21]. Copyright 2018, Nanoscale.

In recent years, flexible and transparent electronic devices have attracted great interests in scientific research and industry with applications in liquid crystal display [1], wearable electronic device [2], electroluminescent devices [3] and solar cells [4], as shown in Figure 1. The transparent conductive electrode (TCE), an important and critical issue in flexible transparent electronic device, has a large influence on the performance of device. At present, the widely used flexible TCE is indium tin oxide (ITO), a transparent conductive film, which is the most representative TCE because of its excellent electrical and optical performances [5,6,7,8]. However, the preparation of ITO film is comparatively complicated and costly. The resource of indium is rare and ITO has low toughness and fracture strength. Therefore, the development of ITO-based TCE is greatly limited, and researchers have investigated suitable alternatives to ITO recently, including graphene [9], carbon nanotubes (CNTs) [10], PEDOT:PSS [11], metal nanowires (NWs), and metal nanostructure networks [12]. Among them, silver (Ag) NWs have received extensive attentions due to their excellent electrical and optical performances. Ag NWs have high mechanical flexibility and high resistance to bending, therefore they have important application value. Ag NWs have one-dimensional (1D) architecture with a diameter of 10–200 nm and a length of 5–100 μm [13]. 

In past decade, researchers studied high-quality, well-formed and high-yield Ag NWs, and proposed various synthesis methods of Ag NWs, such as ultraviolet (UV) light irradiation [22], polyol method [23], template method [24] and solvent thermal method [25], etc. For the Ag NWs prepared by the template method, their morphologies and aspect ratios can be effectively controlled by the parameters of template. The UV light irradiation method through UV photoassisted reduction of Ag^+^ can be applied to the preparation of inert metal nanostructures. Both solvothermal and polyol methods could achieve mass production, but the solvothermal process is carried out at high temperature and high pressure, resulting in high cost and limited applications. The polyol preparation process is facile, low-cost and highly efficient, therefore it has gained extensive attentions for the synthesis of Ag NWs. This review will elaborate and summarize several main synthesis methods of Ag NWs, including UV light irradiation, template method and polyol method. Due to the wide applications of the polyol method for the synthesis of Ag NWs, we will focus on this method. The influences of various factors on the preparation process are analyzed in detail, including the effects of AgNO_3_, polyvinyl pyrrolidone (PVP) and other factors on the morphology and aspect ratio of Ag NWs. Based on the well-formed Ag NWs, the TCE with high conductivity and high transparency is obtained, and this review will introduce several important preparation methods of TCE, such as spin coating [26,27], inkjet printing [28], and roll-to-roll (RTR) printing process [29,30]. On this basis, some common problems in the preparation process are discussed including high junction resistance [31], poor adhesion between Ag NWs and substrate [32], poor thermal stability and environmental stability of Ag NWs TCE [33]. These problems limit the production and applications of Ag NWs TCE. This review will also address these important issues according to the reported research results. Finally, the practical applications and the prospects of Ag NWs TCE in flexible energy storage equipment [34], electroluminescence, electrochromic (EC) devices [35], solar cells [36], thin-film heaters (TFHs) [14] and stretchable devices [21] are summarized.

## 2. Synthesis of Ag NWs 

Researchers have proposed a number of methods for synthesizing nanostructures in last decade, mainly divided into physical methods and chemical methods [37]. The physical methods are mainly based on the mechanical pulverization, and the particle size of the material is generally reduced by means of high-energy ball milling and supersonic airflow pulverization [38], etc. However, the impurities are always introduced into the apparatus during pulverization process, so Ag NWs with high performance are usually synthesized by chemical methods [39]. The chemical methods have the characteristics of facile process, convenient operation, and are easy to scale up, including the template method [40], polyol method [41], photoreduction method [42] and solvothermal method [43], etc., and these methods are summarized and discussed in this review.

### 2.1. Ultraviolet Irradiation

The UV irradiation method is a kind of photoreduction method [44]. For this method, Ag^+^ serves as the precursor solution, and a suitable surfactant is added as the protective agent to induce photoreduction of silver nitrate (AgNO_3_) by a photodecomposition step under UV irradiation conditions. The formation mechanism of Ag nanostructures is described in the following Equation (1):

(1) $$2{\mathrm{AgNO}}_{3}\overset{\mathrm{hv}}{\to }2\mathrm{Ag}+2{\mathrm{NO}}_{2}↑+O_{2}↑$$

**Figure 2:**
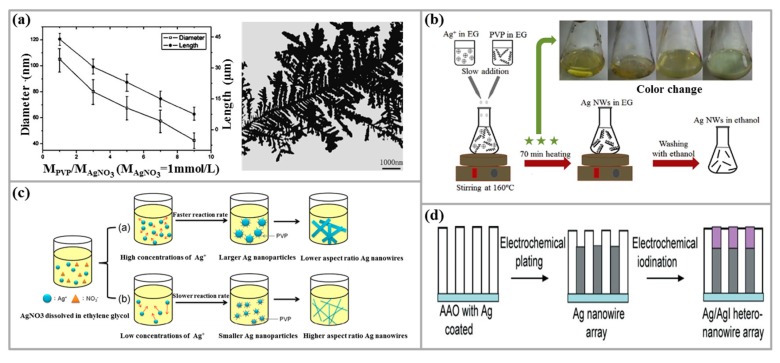
Synthesis of Ag NWs and some influencing factors: (**a**) Diameters and lengths of the Ag NWs as a function of the molar ratio of PVP/AgNO_3_ and transmission electron microscope (TEM) image of Ag nanostructures obtained with 0.5 mL of Ag seeds added [45]. Copyright 2004, Crystal Growth. (**b**) Schematic representation of the polyol synthesis of Ag NWs [58]. Copyright 2016, Materials Chemistry and Physics. (**c**) Schematic of the different concentrations of AgNO_3_ for synthesis of Ag NWs with different diameters [54]. Copyright 2014, Solid State Chemistry. (**d**) Schematic illustration of template synthesis procedures for obtaining the AgI/Ag heterojunction structures [84]. Copyright 2007, Advanced Functional Materials.

Many researchers have synthesized Ag nanostructures by UV light. Among them, Zou et al. [45] developed a method for synthesizing Ag nanostructures and polymer/Ag nanocomposite with polymer surfactants by photochemical reduction and seed-mediated methods. It is found that the molar ratio of PVP/AgNO_3_, the volume of Ag seeds, the presence of PVP and the irradiation time of UV light have important effects on the morphology and aspect ratio of prepared Ag NWs, as shown in Figure 2a. In this study, a conclusion is proposed that only the reactants in contact with UV light can be triggered and reduced to the final product, and this formation mechanism can be applied in fabrication of nanomaterials using shaped molds.

In addition, Zhou et al. [46] studied a novel UV irradiation photoreduction technique for preparing single crystals, and prepared Ag nanorods and dendrimer supramolecular nanostructures by using polyvinyl alcohol (PVA) as the protective agent at room temperature. The concentrations of AgNO_3_ and PVA have significant effects on the formation and growth of these novel nanostructures. For example, as the concentration of AgNO_3_ increases, the Ag nanorods become thick and long, reaching a length of 1 mm and a width of 40 nm under certain parameters. This method can be applied in the preparation of noble metal-based nanostructures. 

For the synthesis of Ag NWs, Liu et al. [47] designed and investigated a green method, utilizing phosphomolybdic acid as catalyst and stabilizer to prepare Ag NWs by UV light irradiation and reduce Ag^+^ in solution. Temperature, UV irradiation time, and molar ratio of AgNO_3_ to phosphomolybdic acid have significant influences on the morphology and aspect ratio of Ag NWs. This study verified that Ag NWs have good antibacterial performance, providing a research basis for antibacterial applications of Ag NWs. 

### 2.2. Polyol Method

Among various ways of synthesizing Ag NWs, the polyol method has certain advantages in cost and mass production, currently the common preparation method of Ag NWs [48]. In 1989, Fievet et al. [49] first reported the polyol method for synthesis of colloidal particles of metals and alloys. By polyol method, an inorganic salt is reduced at a high temperature from a polyol. In an extensive study on the preparation of Ag NWs from polyols [50,51], ethylene glycol (EG) is generally used as a solvent and a reducing agent, PVP is employed as a coating agent and a template, and AgNO_3_ is utilized as Ag source. The synthesis of Ag NWs by polyol method is achieved by other additives, changing the content or concentration of each material, adjusting the temperature and stirring rate [52]. The effects of these parameters on the synthesis of Ag NWs by polyol method are reviewed as follows.

#### 2.2.1. Effects of AgNO_3_

For polyol method, AgNO_3_ plays a very important role as Ag source. The concentration and addition rate of AgNO_3_ will affect the morphology, aspect ratio and yield of Ag NWs [53]. At present, in the study of the influence of AgNO_3_ concentration, it is found that AgNO_3_ concentration is closely related to PVP concentration. There are two main methods for studying the effect of AgNO_3_ concentration, one is that the amount of PVP is constant, and the other is that the molar ratio of PVP/AgNO_3_ is constant [13]. However, most of the researches are performed according to the first method.

Lin et al. [54] studied the effect of AgNO_3_ concentration on the aspect ratio of Ag NWs by polyol method. In their synthesis, the effect of AgNO_3_ concentration on the morphology of Ag NWs is investigated by changing the concentration of AgNO_3_ without changing the PVP concentration, as shown in Figure 2c. Their experimental results showed that the growth of Ag NWs is influenced by synthesis temperature, AgNO_3_ concentration and the rate of adding AgNO_3_. High-aspect-ratio Ag NWs can be efficiently synthesized by reducing the molar concentration of AgNO_3_ without other metal salts as seed source. A study by Coskun et al. [55] also reported that the low molar ratio of PVP/AgNO_3_, the larger diameter of the synthesized Ag NWs. As the molar ratio of both increases, the diameter of the Ag NWs decreases. 

The above study examined the effect of AgNO_3_ concentration without other metal salts in the synthesis. Furthermore, some researchers also studied the change in AgNO_3_ concentration after adding other additives. Amirjani et al. [56] studied the effect of addition rates of AgNO_3_ and Cl^−^ on the preparation of Ag NWs via polyol method by response surface analysis. In the second-order analysis, the addition rate of AgNO_3_ has a greater effect on the reaction than that of Cl^−^. However, when the Cl^−^ concentration is lower than 1.05 mM, the synthesized Ag NWs have a low aspect ratio regardless of the addition rate of AgNO_3_. By adjusting the Cl^−^ concentration to less than 2 mM and the addition rate of AgNO_3_ to less than 0.047 mm/min, the optimal value of the aspect ratio of uniform Ag NW is obtained. Luo et al. [57] observed the morphology change of Ag nanocrystals by adjusting the concentration of HNO_3_, and found that Ag nanocrystals with different morphologies are synthesized by reducing the concentration of AgNO_3_. 

Nekahi et al. [58] also found that the concentration of AgNO_3_ is an important factor in increasing the yield of Ag NWs. In their synthesis, when the AgNO_3_ concentrations are 0.07 M and 0.1 M, respectively, Ag nanoparticles (NPs) and Ag NWs are produced, thereby reducing the yield of Ag NWs. As the concentration of AgNO_3_ increases from 0.07 M to 0.085 M, high-aspect-ratio Ag NWs are formed, and their increased diameter is also achieved (the diameter increases due to the increase in the amount of precipitated Ag atoms). With the AgNO_3_ concentration of >0.1 M, the length of the Ag NWs becomes low. The synthesis principle of Ag NWs by polyol method is introduced, as shown in Figure 2b. Therefore, depending on the synthesis conditions, adjusting the concentration of AgNO_3_ will increase the yield of Ag NWs.

It is concluded from the above research resutls that for the polyol method, the AgNO_3_ concentration has an important influence on the aspect ratio and yield of Ag NWs. By adjusting the concentration of AgNO_3_, Ag nanostructures with optimized performances can be obtained to meet the application requirements.

#### 2.2.2. Impact of Polyvinyl Pyrrolidone 

Due to the structure and properties of PVP, it plays an important role in the synthesis of Ag NWs. On the one hand, the long molecular chains of PVP can be tightly coated on the Ag’s surface to prevent further agglomeration of Ag particles. On the other hand, PVP can specifically be adsorped on the specific crystal face of Ag NPs and reduce their surface activation energy, slowing their growth rate, inhibiting the growth and finally forming Ag NWs with 1D architecture. Therefore, in the synthesizing process of Ag NWs, PVP is indispensable, therefore PVP is studied in many related researches for its influence on the morphology of Ag NWs.

Lin et al. [59] found that both the molecular weight and concentration of PVP pose the effect on the growth characteristics of Ag NWs. In this research, the morphologies of Ag nanostructures are compared with the molar ratios of PVP/AgNO_3_ (0.5, 1.5 and 2.5) by using PVP with molecular weight (M_w_) of 40 K and 360 K, respectively. It is found that the Ag nanostructures involved in the synthesis using PVP with a 40 K Mw are Ag NPs, regardless of the molar ratios of PVP/AgNO_3_ (0.5, 1.5 and 2.5). However, when the M_w_ of PVP is 360 K and the molar ratio of PVP/AgNO_3_ is 2.5, Ag NWs (average length is 20 μm, average width is 170 nm) are synthesized without other metal salts. As the molar ratio of PVP/AgNO_3_ decreases to 1.5 and 0.5, the aspect ratio of Ag NWs tends to decrease gradually, and Ag NPs are also grown. This research work showed that by increasing the molecular mass of PVP appropriately, Ag NWs are efficiently synthesized. 

Yang et al. [60] also found the similar results with those of Lin et al., in the synthesis of Ag NWs by polyol method. In their study, the molecular weight (M_w_) are 24 K, 45–55 K, 58 K and 130 K, respectively, observed by changing the molecular mass of PVP. PVP with larger molecular weight is beneficial to the formation of Ag NWs along their linear growth. The effect of PVP concentration on the morphology of Ag NWs is studied. It is found that as the PVP concentration increases and the molar ratio of PVP/AgNO_3_ increases from 3.0 to 5.0, the length of Ag NWs increases, the width of Ag NWs decreases first, and the aspect ratio of Ag NWs decreases. Because Ag ions will produce crystal nuclei and continue to grow with low PVP concentration. Lack of PVP inhibits the growth of some nuclei, and then some nuclei are grown on larger Ag particles, resulting in a wide Ag NW. As the concentration of PVP increases, the inhibition capacity becomes higher, so the width of Ag NWs decreases. However, with ultra-high concentration of PVP, the molar ratio of PVP/AgNO_3_ will increase from 5.0 to 6.0. After the production of Ag NWs, the low concentration of Ag^+^ can also be restored, so the Ag NWs become wider. This result is also confirmed in the study by Zhang et al [61]. However, as the molar ratio of PVP/AgNO_3_ continues to increase to 8.0 or 10.0, the width of the Ag NWs will suddenly increase by more than 250 nm, and even the aspect ratio becomes low and high-density Ag nanorods are synthesized.

Therefore, in the synthesis of Ag NWs, the concentration and molecular weight of PVP should be adjusted according to the morphology and aspect ratio of Ag NWs required for practical applications.

#### 2.2.3. Addition of Metal Salt

In the synthesis process of Ag NWs by polyol method, except PVP and AgNO_3_ playing a vital role, the addition of some ions will affect the yield and morphology of Ag NWs, so some metal salts are added in the synthesis. For adjustment, several commonly employed metal salts including NaCl, KBr, CuCl_2_, etc., will provide Cl^−^, Br^−^, Cu^2+^, etc., for the synthesis of Ag NWs.

(1) Effect of Cu^2+^

Wang et al. [62] investigated the relationship between Cu^2+^ and morphology of Ag NWs through experiments, that is, the higher the concentration of Cu^2+^, the longer the length of Ag NWs. Since the presence of Cu^+^ (reduced by Cu^2+^) will increase the absorption rate of Ag atoms, the diameter of Ag NW increases from 31 nm to 57 nm, which can also be adjusted by simply controlling the concentration of Cu^2+^ during the reaction. This rapid method can obtain increased productivity of Ag NWs. Chen et al. [63] conducted a study on the crystal quality of Ag NWs by adding two kinds of mediators, CuCl_2_ and FeCl_3_ composed of the same Cl^−^ ion during the polyol process. It is found that the suitable addition of Fe^3+^ ions provides a more favorable method for obtaining Ag NWs with high crystallinity and uniform size. 

(2) Effect of Halide Ion

Kumar et al. [64] added NaCl during the synthesis of Ag NWs by polyol method, and AgCl is formed by Cl^−^ and Ag^+^ to control the concentration of Ag^+^ in the solution during the initial formation of Ag seed, and then Ag NWs with various directions are synthesized under the action of PVP. The effectiveness of Ag NWs in transparent flexible devices is fully verified for heterogeneous Ag NWs with a diameter of ~50–80 nm and a length of ~5–30 μm. Li et al. [65] synthesized Ag NWs with a diameter of ~100 nm and a length of several micrometers with the aid of PVP and NaCl by polyol method. It is indicated that the Ag NWs are preferentially formed at a higher NaCl concentration. Similarly, Trung et al. [66] also found that Ag NWs became longer and thinner by increasing NaCl concentration, and Ag NPs are formed without NaCl. They also added 0.4 mM KBr in the solution and found that the diameter of Ag NWs further decreased to 20–50 nm and the length increased to 30–60 μm. The results indicated that proper mixing of metal salts is the key to the synthesis of ultrathin Ag NWs. Zhang et al. [67] conducted a series of experiments to study the effect of halide ions on the morphology of Ag NWs. In their synthesis, the total amount of halide ions is constant, and only the ratio of chloride ion to bromide ion is changed. As KBr content increases, the diameter of Ag NWs decreases first and then increases, and the length of Ag NWs decreases. This study facilitates the synthesis of size-controlled Ag NWs by bromide-mediated polyol method. Silva et al. [68] used a one-pot polyol method to synthesize high-quality Ag NWs with a diameter of less than 20 nm and an aspect ratio of more than 1000. This method has the advantages of simplicity, high stability, and fast acquisition. Br^−^ (1,300,000 g/mol) and PVP play a key role in the lateral inhibition of Ag NWs, and high-aspect-ratio Ag NWs are obtained. 

#### 2.2.4. Other Factors

Except the effects of AgNO_3_, PVP and other metal salts, there are other influencing factors such as synthesis temperature, stirring rate, injection time and microwave assist of the catalyst in the synthesis of Ag NWs by polyol method.

(1) Temperature

Many researchers have verified that temperature poses the effect on reducing ability of Ag NWs during synthesis, and the reducing power increases with ambient temperature increasing. This effect is due to the temperature dependence of EG oxidation to form the reducing agent of acetaldehyde. Moreover, a high yield of Ag NWs is obtained by using a heating process in the polyol. Xia et al. [69] showed the Ag nanostructures synthesized at different reaction temperatures and found that only Ag NPs are formed at 100 °C for a long time. Because the relatively low reaction temperature does not seem to provide sufficient energy for anisotropic growth of Ag NWs. When the temperature increases to 160 °C, it is found that well-formed Ag NWs are synthesized. The test results from Sun et al. [70] also showed that the length of Ag NWs decreases significantly when the reaction temperature is higher or lower than 160 °C. Nekahi et al. [58] also studied the effect of temperature on the aspect ratio and yield of Ag NWs at 150 °C, 160 °C and 170 °C. The results showed that the Ag NWs with a longer length and a higher aspect ratio are produced at 160 °C. On the other hand, higher yields of Ag NWs are obtained at higher temperatures. 

(2) Others

In the synthesis of Ag NWs, it is found that by controlling the stirring time, injection time and microwave assist of the catalyst, the morphology of the Ag nanostructures can be appropriately tuned. For example, A. Gómez-Acosta et al. [71] adjust the Ag NWs synthesized by polyol method. Other factors are remained the same. By controlling the stirring time of PVP before adding the salt, the scanning electron microscope (SEM) characterization results showed that the longer the stirring time, the more obvious the linear structure of Ag. Lin et al. [72] studied the effect of light irradiation on the morphology and yield of Ag NWs during the synthesis by polyol method. It is found that Ag NWs with good morphology are synthesized when the reaction suspension is irradiated with light of 400–500 nm wavelength at the nucleation stage, it will produce high yield. The results showed that light can accelerate the formation of NP seeds that are most suitable for the growth of Ag NWs. Yi et al. [73] synthesized high-quality, high-yield Ag NWs by microwave-assisted polyol technology under the premise of adding NaBr solution, and this method can adjust the diameter of Ag NWs by controlling microwave power and concentration of NaBr solution, which is a large-scale synthetic diameter, and this provides a new way for the preparation of Ag NWs. 

### 2.3. Template Method

The template method not only tunes the shape of the final product by controlling the shape of the template, but also is an important way for preparing regular nanomaterials. This method is mainly divided into the hard template method and soft template method [74]. The hard template method is mainly divided into anodized aluminum oxide (AAO) template method and CNT template method, etc. The soft template method is mainly divided into biomolecular template method and ionic liquid template method, etc. [75,76,77]. The stencil method has the advantages of being able to easily synthesize aligned NWs array, and low cost of the template, but it also has the disadvantages that the post-processing is complicated and it is difficult to achieve mass production. This section will introduce several template methods.

#### 2.3.1. Hard Template Method

A hard template refers to a rigid template that maintains a specific shape by covalent bonds. For example, the polymer having a different spatial structure, AAO film, porous silicon, CNTs, etc. The advantage of the hard template method is the easy control. The synthesized Ag NWs are highly ordered, uniform and adjustable in size, and the agglomeration between Ag NWs is hindered [78]. However, the hard template complicates the purification process of Ag NWs, and causes damage to the NWs simultaneously, especially to the high-aspect-ratio Ag NWs during the purification process. Among them, porous AAO template is one of the effective methods for preparing 1D nanomaterials due to its high reliability, high mechanical strength, good insulation, facile preparation, high order and controllable pore size. 

(1) Anodized Aluminum Oxide Template Method

AAO consists of an outer thick porous layer and a tight barrier layer adjacent to the aluminum substrate. In the 1990s, with the rise of self-assembled nanostructures [79], nanomaterials with highly ordered nanoscale array channels have received much attention [80]. Dan et al. [81] studied the morphology of pyrogenic AgNO_3_ synthesized Ag NWs at different temperatures by using the AAO template method, and the specific preparation process is proposed. Their results showed that the Ag NWs are synthesized in the nanopores of AAO template. The molar ratio of PVP/AgNO_3_ is 8/1, the constant EG concentration is 6ml/l, the temperature is 170 °C, the pyrolysis time is 60 min, and the diameter is finally obtained as about 70–80 nm, with Ag NWs of about 2 microns in length. Song et al. [82] synthesized Ag NWs by solution thermal method using AAO template method as an auxiliary method. By suspending the AAO template deposited with Ag NWs in rhodamine B (RhB) solution, it is found that the uneven surface of the porous AAO substrate resulted in the formation of a series of gaps between Ag NWs and fluorescent molecules of less than 10 nm, which is effective in enhancing the fluorescence of the RhB solution. It is indicated that the AAO template has great potential for fluorescence-enhancing dye molecules, which is of great help for the selection of metal-enhanced fluorescent molecular substrates. Arefpour et al. [83] proposed a high-throughput method for the synthesis of ultra-long Ni, AgNi, AgCu and AgNiCu NWs with high uniformity using metal ion electrodeposition into an AAO template. This method could achieve reduced cost of using electrochemical and self-assembled nanopores, making the electrochemical deposition method based on AAO templates more attractive. Liang et al. [84] also prepared AgI/Ag heterojunction NWs using the AAO template method, and the specific preparation process is shown in Figure 2d. Wang et al. [85] reported the fabrication of vertically placed Ag nanosheets and Ag NWs directly on ITO TCE by AAO template method. In this study, the AAO film was prepared directly on the substrate by anodization to complete the AAO template demolding process. This method for preparing NWs can be applied to other metals on various conductive substrates, and has broad application prospects. 

#### 2.3.2. Soft Template Method

The soft template method refers to a cluster of templating agents that form a certain spatial structural feature by non-covalent bond forces, such as intermolecular or intramolecular weak interactions. Such clusters collectively have a distinct structural interface, and the distribution of inorganic substances exhibits a specific tendency to obtain nanostructures with specific structures through this unique structural interface. The soft stencil method has the advantages of morphological diversification, easy construction, and no need for complicated equipment, but this method has low structural efficiency due to poor structural stability. The soft template method mainly includes a DNA template method and an ionic liquid template method.

(1) DNA Template Method

DNA is composed of a large number of deoxyribonucleotides, has strong molecular recognition ability and self-assembly ability, and can regulate its self-assembly by controlling shape, length and sequence. Chen et al. [86] prepared a Ag nanochain by assembling Ag ions on the DNA strand, and Ag ions are reduced by tannic acid. Cui et al. [87] used electrochemical methods to assemble Ag ions onto a DNA template and reduce metal ions to prepare Ag NWs with a diameter of about 50 nm and a length of about 6.0 μm. The presence of DNA is the most important condition for the synthesis of Ag NWs in this method. The Ag NPs aggregate along the DNA strand. Without the DNA strand, the Ag NPs will be in a dispersed state. Moreover, the length of the DNA molecular chain controls the length of the Ag NWs.

(2) Ionic Liquid Template

Due to its electrochemical stability, wide electrochemical window and high electrical conductivity, ionic liquids (ILs) have good ionization and high electrical conductivity, and can act as stabilizers on the surface of Ag NPs. It has a good dispersion to prevent the agglomeration of Ag NPs. Kim et al. [88] investigated the formation of Ag NWs by IL method. The results showed that the morphology of the obtained Ag NWs is obviously related to the stability and self-organization of ILs, so they can be adjusted based on the type and reaction conditions of ILs. 

## 3. Preparation of Ag NWs Transparent Conductive Electrodes

Two preparation methods are commonly used for synthesis of Ag NWs, one is the “subtractive” preparation, and the other is “addition” preparation. For this “addition” preparation, the Ag NWs are formulated into ink, and then they can be used. Environmentally and economically friendly printing methods, including inkjet printing, screen printing, gravure printing, RTR process, etc., or using drop coating, spin coating, etc., to deposit Ag NWs ink onto the surface of the substrate. At the beginning, the photolithography method is mainly employed for the preparation of transparent electrodes, which has good fidelity, can effectively reduce surface roughness, and is compatible with flexible substrates. However, photolithography is expensive, and the process including photolithography, development and rinse is complicated. With the increasing awareness of environmental protection and the deepening of research, more and more researchers are adopted the “addition” preparation method. The preparation process of the drop coating is facile and the demand for the Ag NWs ink is low, but the surface of the obtained Ag NWs electrode is particularly rough, and the Ag NWs are difficult to distribute uniformly. Compared with the drop coating, the Ag NWs ink can be dispersed on the substrate and form a uniform Ag NWs film under the action of centrifugal force during the spin-coating process, and the obtained surface is relatively flat, which can achieve low cost and high efficiency. Now the spin-coating method is also a major preparation method for preparing Ag NWs TCE. However, it is difficult to achieve large-volume, large-scale production by spin coating [89]. With the development of the printing process in electronic devices, more and more researchers have begun to study and use the printing method to prepare transparent electrodes. However, the Ag NWs TCE prepared by spin coating or printing process has several problems, such as high junction resistance, poor adhesion to the base, poor mechanical stability, poor thermal stability, etc. The process most likely to achieve mass production is the RTR process. This section first introduces the research on the preparation of transparent electrodes by spin coating, inkjet printing technology and RTR process, and then the current research state and the common problems of these three methods will be discussed.

### 3.1. Preparation Method

#### 3.1.1. Spin-Coating Method

For spin coating method, the main equipment is the homogenizing machine. The spin-coating method has three steps of batching, high-speed rotation and volatilization into film, by controlling the time of the glue, the rotation speed, the amount of the liquid and the solution used. The concentration and viscosity are important parameters for controlling the thickness of the film.

Among them, the rotational speed has a great influence on the thickness and performance of film. For the same material, the higher the speed, the thinner the film when rotating at high speed; the lower the speed, the thicker the film when rotating at high speed [90]. However, when the angular velocity of the high-speed rotation is too fast, the film is easily broken, whereas the angular velocity is too low, the Ag NWs in the ink are liable to aggregate, resulting in uneven distribution of the Ag NWs. The higher the rotation speed, the smaller the number density of Ag NWs, the higher the transmittance of the electrodes, and the worse the conductivity. Therefore, the setting of the angular velocity of high-speed rotation is very important for the performance of the electrode. Ag NWs TCE with different transmittances can be obtained by controlling the rotation speed of spin coating.

**Figure 3:**
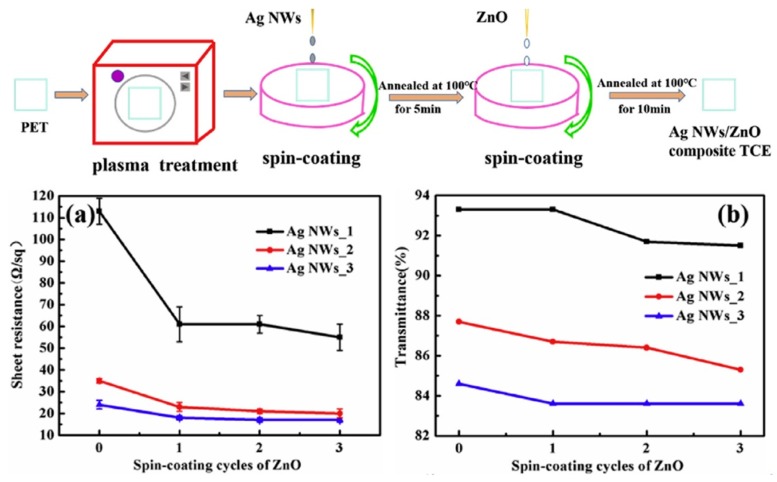
The schematic illustration of the preparation of Ag NWs/ZnO composite TCE and the sheet resistance, transmittance at 550 nm and figure of merit for the Ag NWs TCEs with different spin-coating cycles of ZnO, respectively [91]. Copyright 2018, Journal of Alloys and Compounds.

Tang et al. [91] adjusted and optimized the density of Ag NWs and the thickness of ZnO NPs through different spin cycle cycles in low-temperature environments (maximum temperature < 100 °C). It is found that when Ag NWs is cycled once, the sheet resistance and transmittance of TCE are 113 Ω/sq and 93%, respectively. When Ag NWs is cycled for 2 cycles, the sheet resistance and transmittance are reduced to 35 Ω/sq and 88%, respectively, indicating that the sheet resistance of Ag NWs/ZnO composite increases with the spin-coating period of Ag NWs. The resistance is reduced, and the transmission rate is also reduced. However, the finally prepared Ag NWs/ZnO TCE has excellent flexibility, environmental and thermal stability (~300 °C), high electrical conductivity (~20 Ω/sq) and good optical transparency (about 87% at 550 nm), as shown in Figure 3. Therefore, Ag NWs TCE with different sheet resistances and transmittances can be obtained by controlling the spin cycle. 

In addition, there is a concern in the spin coating of Ag NWs that they are randomly oriented, and sometimes the randomly oriented Ag NWs will form so-called dead spots on the device. These Ag NWs typically form gaps that cannot be filled with mesoporous material. This is detrimental to the effective charge transfer from the device to the grid. Fortunately, this problem can be solved by filling a layer between the NWs that does not block most of the incident light conductive fill layer. The presence of conductive fillers allows carriers to transfer charge to the nearest NW with minimal resistive losses, studied by Alami et al. [92] who filled ZnO as a conductive filler and the series resistance caused by lateral transport of carriers between Ag NWs is reduced. Moreover, Ag NWs/ZnO TCE could obtain improved effective electron mobility and electrical conductivity, and the mechanical and thermal stability of TCE is significantly improved. The related issues have also been addressed in the study by Ricciardulli et al. [93]. 

Although the spin-coating method currently occupies a large proportion in the preparation of Ag NWs TCE, it cannot directly realize patterning. For electrodes with high performance and fine structure, an additional etching process is required to meet the demand, so the preparation of Ag NWs TCE by spin coating still has problems and can be widely used in research or experiments, however it is difficult to achieve large-scale, high-volume production.

#### 3.1.2. Inkjet Printing 

Inkjet printing is a technique that accurately deposits functional materials on corresponding parts of a substrate by means of contactless, pressureless, and non-printing, and is an effective way to prepare TCEs. Inkjet printing technology is a technique in which a conductive ink is dispersed by dissolving a conductive material such as Ag NWs in a corresponding solvent, and then ink is ejected under a computer control to form a dot pattern [94]. At present, inkjet printing technology is mainly divided into continuous inkjet and drop-on-demand inkjet, and the continuous inkjet is earlier and faster than the drop-on-demand inkjet printing. However, the continuous inkjet printer adapts to faster printing speed, and it is required to be equipped with a deflection device and an ink droplet charging device, etc., which increases the cost and its ink utilization rate is low [95]. Currently, drop-on-demand inkjet printing technology is commonly used to prepare Ag NWs TCEs at a lower cost and with flexibility. Compared with the spin coating method, inkjet printing is suitable for automation and high-resolution patterning, and multiple ink cartridges can be used to deposit different materials simultaneously. The content of each material can be precisely controlled. The direct access to the patterned TCE simplifies the process and reduces the cost, and further promotes the application of Ag NWs TCE and other conductive metal NWs TCEs. Therefore, in recent years, many researchers have conducted related research.

Lu et al. [96] developed a variety of printing methods represented by inkjet printing, which can directly obtain a patterned transparent conductive film, and achieve a good effect of a transmittance (more than 85%) and a square resistance of less than 20 Ω/sq. The inkjet-printed Ag NWs are successfully implemented as the top transparent electrode of the translucent organic photovoltaic cell (OPV). This device achieves translucency and reduces electrode preparation cost, while the conversion efficiency of the device is also very good. 

**Figure 4:**
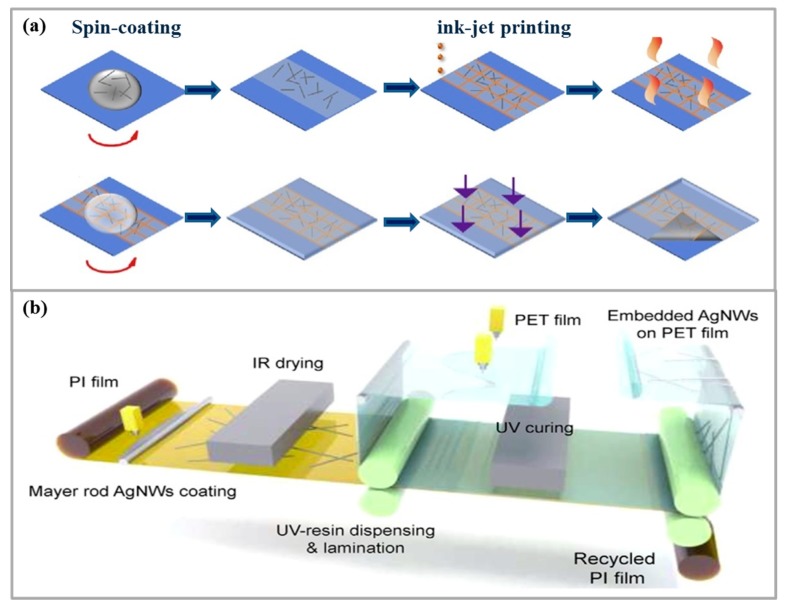
Two preparation methods of Ag NWs TCE. (**a**) Fabrication processes of the hybrid electrode on a photopolymer substrate [98]. Copyright 2017, Organic Electronics. (**b**) Schematic illustration of the RTR fabrication process for the embedded Ag NWs TCE on PET film [102]. Copyright 2017, Organic Electronics.

Although TCEs based on Ag NWs already have high performance, the looseness and local insulation problems caused by the gaps between NWs still limit their applications in electrodes. Kinner et al. [97] used inkjet printing to fabricate Ag NWs based TCE. In their research, embedded and non-embedded Ag grids are used to prepare polymer light-emitting diodes (PLEDs). For the fabrication process, an Ag grid was printed in a honeycomb layout by inkjet, and then coated with high-conductivity PEDOT:PSS on the grid. Finally, in order to further improve the efficiency of the device, a honeycomb Ag grid is embedded in a material based on OrmocerVR, which reduces leakage current and enhances optical coupling. The results showed that inkjet printing of ITO-free embedded Ag-PEDOT:PSS PLEDs significantly improves efficiency by 250% compared with ITO-based PLEDS. Tao et al. [98] also conducted a corresponding study to prepare a transparent hybrid electrode by using the Ag grid and Ag NWs. The Ag grid printed on the Ag NWs film is used to connect the gaps between Ag NWs to increase the overall conductivity. This hybrid electrode has a low resistivity (22.5 Ω/sq) while maintaining high transmittance (87.5%), which is comparable with ITO-based TCE, and its preparation process of spin coating is shown in Figure 4a. 

Ag NWs TCE can be obtained quickly, at low cost and in large quantity by inkjet printing. However, some studies found that Ag NWs TCE has lower electrical and optical performances when subjected to external mechanical damage. In order to solve this problem, hard materials are deposited. The Ag NWs network is widely used, but its brittleness limits its wide range of applications. Li et al. [99] printed a mechanically flexible and robust 5-μm acrylic polymer-silicate NP composite resin on Ag NWs TCE by an electrohydrodynamic (EHD) inkjet printing process during the preparation of Ag NWs TCE. The combined device has a transparency of 90% and a sheet resistance of 45 Ω/sq, and its flexibility and mechanical stability are greatly enhanced. For inkjet printing technology in the preparation of Ag NWs TCE, not only the preparation of Ag NWs film but also the preparation of protective layer, can be carried out. Moreover, by improving the inkjet printing technology, the EHD jet printing process, as an emerging process, can produce a high-resolution pattern with fine droplets under an applied electric field, further improving printing accuracy.

#### 3.1.3. Roll-To-Roll Process 

Currently, the process most likely to achieve mass production is the RTR process. The RTR process is a new process of producing an electronic device by crimping a flexible substrate such as a polymer film or a metal film on a counter roll. The process includes forward/reverse gravure printing, forward/reverse facing rolls, slot die and spray-coating processes. The RTR process enables electronic ink to be applied on a flexible substrate with a few meters wide and 50 m long [100], resulting in a significant reduction in production cost. Its yield greatly exceeds that of the traditional magnetron coating process. Based on the advantages of the RTR process, many researchers have conducted related research.

Kim et al. [101] prepared large-area Ag NWs TCF by continuous RTR slot die coater, and found that different flow resistances (30–70 Ω/sq), light transmittance (89%–90%) and haze (0.5%–1%) of Ag NWs TCF can be obtained by controlling the flow rate of conductive Ag NWs inks, and it has high mechanical stability and flexibility. Due to its excellent performance and the large-scale preparation advantage of RTR, Ag NWs TCF can be used as a viable alternative to traditional ITO electrodes for cost-effective and large-area flexible touch screen panels (TSP). Jung et al. [102] successfully fabricated Ag NWs TCEs on polyethylene terephthalate (PET) film substrates by RTR process, and then large-area Ag NWs TCEs by RTR vacuum deposition process, as shown in Figure 4b. The OLED is deposited directly on the embedded Ag NWs electrode. OLEDs with embedded Ag NWs electrodes on PET films exhibit 30% to 40% higher performance than that of ITO TCE OLEDs, and have a sheet resistance of only 5 Ω/sq and an optical transmission of 85%, with excellent electrical and optical properties. The performance provides a reliable basis for the preparation of TCEs for large-area flexible OLEDs. At room temperature, Kim et al. [20] prepared high-performance, flexible and transparent EC films by RTR sputtering on ITO/Ag/ITO (IAI) multilayer electrodes. The IAI multilayer electrode sputtered on PET substrate exhibited a high optical transmittance of 82.4% and a low sheet resistance of 8.93 Ω/sq. Based on the EC performance, the RTR-sputtered IAI multilayer enables large-area, transparent, flexible EC devices for flexible smart window applications. 

The RTR process has outstanding advantages in the preparation of large-area TCE, and the current RTR process still involves vacuum deposition, sputtering, etc. In the development of large-area TCE, the RTR process still needs to be improved.

### 3.2. Solution

Although the above three methods have been widely used in the preparation of Ag NWs TCE, Ag NWs have some defects, limiting their large-scale commercial applications, which are summarized as the following points: (i) Ag NWs cross-connection formation causes the rough film/electrode surface, and protruding nanostructures easily form current channels through the device, resulting in shunting or short-circuiting; (ii) poor adhesion between Ag NWs and the substrate, and poor mechanical stability; (iii) thermal stability and environmental stability of Ag NWs TCE are poor. In order to solve the above problems, researchers have made great efforts, and this section will review the improvement proposals by researchers in the past decade for the aforementioned defects of Ag NWs.

#### 3.2.1. High Junction Resistance

Although Ag NWs are often used to prepare flexible electronic devices, their surface roughness and high junction resistance hinder the realization of high optoelectronic performances of devices. For example, high-performance OLED devices require flat and smooth electrode surfaces to achieve uniform electric field and charge. The carrier is implanted and uniformly illuminates [92]. The unevenness will cause partial electrical collapse of the device when the surface is not smooth and the electric field strength at the peaks and bumps is higher than that of the uniform layer. Moreover, many of the prepared Ag NWs films suffer from high contact resistance due to the weak contact of Ag nanogaps or junctions. Poor line–line contact also affects the mechanical flexibility of Ag NWs film because loosely stacked Ag NWs tend to move under deformation, resulting in poor conductivity [103]. Therefore, some approaches are required to improve the surface flatness, enhance the line contact between Ag NWs, reduce the junction resistance, and provide a reliable basis for the further development of TCE.

(1) Optimization of Conductive Ink 

Ink is indispensable for spin coating or printing. Ag NWs ink plays a vital role in preparing TCEs by printing process. In order to prepare Ag NWs TCE with high light transmittance and high conductivity, it is necessary to obtain Ag NWs with small diameter and large length. In order to obtain Ag NWs with good morphology, we need to optimize the process parameters of the polyol method. In addition, after obtaining Ag NWs with high aspect ratio, it is necessary to reduce the thickness of PVP coating on the surface of Ag NWs in order to obtain higher performance of Ag NWs ink. PVP is a kind of polymer surfactant. The Ag NWs reaction system prepared by polyol method can be used as dispersing agent to control the size of the product and prevent agglomeration of Ag NWs. When the reaction is completed, a layer of PVP coating of varying thickness is formed on the surface of Ag NWs. PVP is a non-ionic polymer compound that itself is insulated. Therefore, the thinner the coating on the surface of the Ag NWs, the smaller the contact resistance between Ag NWs, and the lower the surface resistance of the electrode. Therefore, if a PVP coating on the surface of the prepared Ag NWs can be thinned by a suitable pretreatment method, the electrical resistance at the contact point can be lowered to improve the overall electrical conductivity of the electrode. Through such a pre-thinning treatment of the PVP cladding layer, the conductivity of the high transmittance region of the entire Ag NWs TCE can be improved effectively.

(2) Other Methods

**Figure 5:**
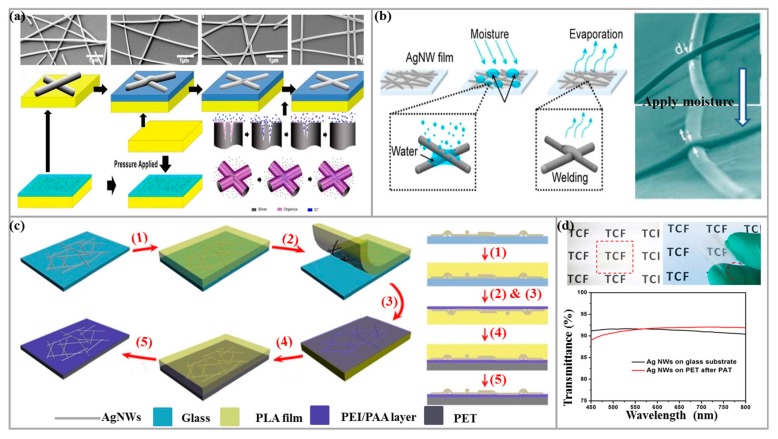
Solutions to the problems of high junction resistance, poor mechanical stability and poor thermal/environmental stability in Ag NWs TCE preparation: (**a**) SEM images of pristine Ag NWs, NaAlg/Ag NWs composite film, NaAlg/Ag NWs composite film after mechanical pressing and NaAlg/Ag NWs composite film after mechanical pressing and CaCl_2_ treatment and the schematic diagrams of junction between Ag NWs corresponding to SEM images, and the schematic illustration of a possible mechanism of crack renovation [104]. Copyright 2017, ACS Applied Materials and Interfaces. (**b**) Schematic of moisture treatment for capillary-force-induced cold welding of Ag NWs [103]. Copyright 2017, Nano Letters. (**c**) The 3D (left) and sectional (right) schematic peel-assembly-transfer (PAT) procedure for the insertion of the PEI/PAA adhesion multilayer between Ag NWs and PET substrates. (**d**) The prepared Ag NWs TCF on PET substrate indicating that the film is transparent and flexible, and the transmittance spectra of the Ag NWs network before and after PAT process [109]. Copyright 2017, Langmuir.

At present, in addition to the pretreatment of conductive ink, a series of follow-up work is performed, including heating, partial welding, mechanical pressing, electrochemical coating, adding materials as soldering agents, etc., to improve wire contact, to ensure smooth surface and reduce the node resistance. However, heating requires precise control of the heating temperature and time, and can affect the use of some heat sensitive substrates or the stability of Ag NWs. The imprinting technique is relatively easy. However, mechanical stamping is not suitable for some devices because high pressure may damage some useful structures or active layers. However, the damage can be repaired to a certain extent by solution treatment [104], as shown in Figure 5a. In addition to mechanical pressing, local welding technology has attracted widespread attentions in recent years, mainly divided into local joule heating welding and plasma induction [105]. Welding, light-induced plasma nano-welding technology, etc., have been widely used in Ag NWs TCE, and experimentally verified that TCE also has high photoelectric performance. However, all of the above methods require chemical reagents or specific equipment to assist in the implementation, and the operation is complicated. In recent years, researchers have proposed some more convenient methods.

Liu et al. [103] found that capillary force is a powerful driving force at the nanometer scale, which can effectively cause self-limiting cold welding of line-wire connections of Ag NWs. Capillary force-induced welding can be easily obtained by applying moisture to Ag NWs film, as shown in Figure 5b. The results showed that the moisture-treated Ag NWs film exhibited a significant reduction in sheet resistance, but the change in transparency is negligible. It also proved that this method can effectively cure the damaged Ag NWs film of the wearable electronic device. Kou et al. [106] proposed a simple method of sun exposure to increase significantly the conductivity of Ag NWs TCE without significantly reducing light transmission. After 1 h or more of sunlight, the Ag NWs network can still achieve a sheet resistance of <20 Ω/sq and a transmittance of ~87% at 550 nm, and excellent mechanical flexibility. Because the sun is completely environmentally friendly and readily available, there is no need for any special post-processing of Ag NWs network TCEs, and any complicated or expensive facilities are not required. It is very economical and convenient, so it is suitable for large-scale applications, especially for outdoor applications. Moreover, the Ag NWs network can be directly integrated without any post-processing and self-improvement in the process of exposing to natural sunlight. This environmentally friendly approach has a very broad application prospect. 

#### 3.2.2. Low Adhesion and Poor Mechanical Stability

Ag NWs flexible transparent electronic devices have wide applications with high electrical conductivity, transparency and flexibility, but the adhesion between Ag NWs/Ag-grid networks and flexible substrates is weak in practical use. It usually reduces the flexibility and stability of the device, and even leads to catastrophic device delamination when the substrate is stretched or twisted, which causes irreversible recovery of TCE, hindering its practical applications.

Kim et al. [107] found that the weak bonds between Ag NWs and polymers limit their adhesion, and strong pulsed light is employed to improve adhesion. Afterward, Kim and other researchers [108] placed a layer of Ag NWs on the surface of the substrate, and then a regular arrangement is formed by illuminating the lamp. A rigid island disk formed by photolithography is placed on it and packaged with a soft polymer. Forming a rigid island disk embedded structure, and a layer of Ag NWs on the surface of the soft polymer, the researchers believe that it may due to the formation of strong covalent bonds between the rigid island disk and the flexible polymer, so the bonding force at the interface is greatly enhanced, thus forming a smooth, transparent, mechanically stable electrode. 

Wang et al. [109] prepared highly transparent, conductive and bendable Ag NWs TCEs with excellent mechanical stability by introducing a polyelectrolyte multilayer (PEI/PAA) between the diol ester (PET) substrates based on a strip-assembly-transfer procedure, and the preparation process and transparency parameters are shown in Figure 5c,d. Lee et al. [110] added encapsulating polymer to strongly adhere Ag NWs and polydimethylsiloxane (PDMS) for Ag NWs/PDMS TCE. 

In summary, by using an embedded structure, adding an adhesive, encapsulating with an encapsulant, adding other solutions to increase cross-linking, etc., the adhesion between Ag NWs and flexible substrate is enhanced, and the mechanical properties of the device is improved. It provides a solid foundation for practical applications of Ag NWs TCE.

#### 3.2.3. Poor Thermal Stability and Environmental Stability

Ag NWs TCE has poor stability, including thermal stability and environmental stability, manifested by aggregation at temperature much lower than the melting point (about 200 °C) and rapid oxidation when exposed to moist air. Moreover, Ag NWS TCE may be subjected to thermal and oxidative stress during post-manufacturing processing and actual operation. In this case, the stability of Ag NWs causes the flexible device to malfunction, which creates great difficulties for practical applications, so the development of Ag NWs TCE with heat resistance and environmental resistance is very urgent.

In recent years, some methods have been proposed to solve this problem, mainly to explore the appropriate materials on the original TCE to continue the packaging process. Some of these studies have proposed the encapsulation of Ag NWs TCE with high melting-point materials, such as sol-gel deposited TiO_2_ films as encapsulants. In addition, Ag NWs TCE is encapsulated by atomic deposition. For example, Yeh et al. [111] used a deposition method to coat the surface of Ag NWs with a highly uniform TiO_2_ as a protective layer to improve the stability of Ag NWs TCE at high temperature and sustainable use. However, in these studies, only the thermal stability of Ag NWs TCE is elaborated, and the environmental stability of Ag NWs TCE is not characterized. Hwang et al. [112] used an atomic layer deposition method to encapsulate an ultra-thin aluminum oxide film (~5.3 nm) on Ag NWs TCE. The experimental results showed that the TCE remains stable even after 100 minutes of annealing at 380 °C. Preventing the penetration of water molecules, the environmental stability can be improved to more than 1080 h in an atmosphere with a relative humidity of 85% at 85 °C, demonstrating its excellent thermal stability and environmental stability. 

## 4. Applications of Ag NWs TCE

In recent years, flexible transparent electronic devices have attracted great interests in research and industry including transparent solid supercapacitors (SCs), OLEDs, superelastic transparent TFHs, solar cells, EC devices, etc. Transparent electrode, especially the flexible transparent electrode is an important part of the device and is attracting widespread attentions. Currently, the widely used flexible transparent electrode is a transparent conductive oxide (TCO), the most representative of which is an ITO TCE, generally produced by magnetron sputtering, e-beam evaporation, chemical vapor deposition, sol-gel, etc. Moreover, ITO has been widely used as a standard transparent film electrode because of its excellent electrical conductivity and optical transparency. However, it is difficult to achieve mass production due to the following shortcomings of ITO: (i) indium is a rare metal, expensive and resource-poor; (ii) ITO has low toughness, high brittleness, poor flexibility and poor adhesion to flexible substrate; (iii) there is a complicated preparation process in which a number of vacuum processing techniques are involved and the film preparation processing temperature is high, which is disadvantageous for substrate with a low melting temperature. Based on the above reasons, the development of ITO TCE has been greatly limited. Therefore, in recent years, researchers have been making great efforts to investigate suitable candidates for ITO, including graphene, CNTs, PEDOT:PSS, metal NWs and networks, and the comparison of properties of these different TCEd are shown in Table 1. Graphene TCE has excellent electrical and optical properties, but its high processing temperature, high processing cost and complicated processing technology hinder its development. CNT TCE has excellent tolerance and flexibility, but its poor conductivity limits its development. The flexible conductive polymer PEDOT:PSS TCE has a limited application range due to poor film stability [113]. In comparison, metal NWs and metal nano networking structures are superior in performance and preparation, such as Ag NWs, Ag nanofibers (NFs) and Ag nano networking structures. Ag NWs has excellent conductivity and optical properties and low preparation cost. The prepared Ag NWs film is flexible and easy for mass production. Because it is comparable with ITO in some properties, it is suitable for candidate of ITO. Therefore, the prepared Ag NWs TCE has been widely used in flexible electronics devices. 

### 4.1. Flexible and Transparent Energy Storage Equipment 

In recent years, flexible transparent electronic devices have attracted wide attentions in many fields. In order to realize low power supply of electronic device, a suitable energy storage device is an indispensable part of flexible transparent electronic devices. The integrated energy storage device further facilitates the use of flexible transparent electronic devices, such as electrical devices that can be attached to the human body, EC smart windows, etc. Ag NWs/Ag-grid TCE is important electrode widely used in transparent energy storage equipment due to its excellent electrical conductivity, optical properties and flexibility, and has attracted special attentions in the development of various electronic and energy storage devices.

**Figure 6:**
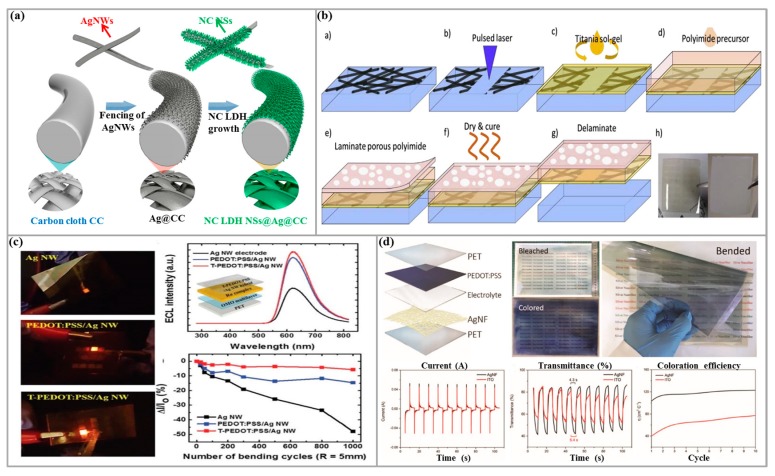
Some applications of the Ag NWs TCE. (**a**) Schematic illustration showing the preparation process of NC LDH NSs@Ag@CC by a single-step electrochemical deposition (ECD) process [115]. Copyright 2017, Nano Energy. (**b**) Preparation of the conductive and scattering flexible substrates [119]. Copyright 2017, Organic Electronics. (**c**) Photographs of the light emission of the Ru-based ECL displays with a Ag NWs electrode (top), PEDOT:PSS/Ag NWs hybrid electrode (middle), and T-PEDOT:PSS/Ag NWs hybrid electrode (bottom) and the ECL spectra of the flexible Ru-based ECL displays with the three types of electrodes and the relative change in the intensity of the flexible Ru-based ECL displays with the three types of electrodes as a function of bending cycles [118]. Copyright 2017, Chemical Communications. (**d**) Fabrication and properties of Ag NFs ECSW [124]. Copyright 2017, Advanced Material.

Xu et al. [114] designed a high conductivity, high transmittance, high mechanical flexibility current collection for high-performance flexible transparent SCs by soft UVnanoimprint lithography (UVNIL) and scraping techniques. The device made of Ag-grid TCE has ultra-high conductivity, optical properties and mechanical stability and flexibility that can withstand repeated bending. Sekhar et al. [115] successfully prepared the binder-free nickel-cobalt layered double hydroxide (NC LDH) nanosheets on Ag NWs-fenced carbon cloth (NC LDH NSs@Ag@CC) by a facile electrochemical deposition, as shown in Figure 6a. The fabricated asymmetric SC (ASC) exhibited a maximum operating potential window of 1.6 V, a high area capacitance of 230.2 mF/cm^2^, and excellent cycling stability of 88.1% under all charge and discharge conditions. The excellent performance of the NC LDH NSs@Ag@CC electrode and constructed ASC has laid a solid foundation for the development of flexible energy storage devices. 

Ag NWs/Ag-grid TCE is not only used in flexible and transparent energy storage equipment, but also can be applied in flexible smart windows combined with energy storage devices and electrochromism to achieve multi functions. For example, Shen et al. [116] prepared Ag NWs/WO_3_ electrode with excellent flexibility and high capacitance. This electrode not only exhibits high coloration efficiency and fast response speed, but also has good electrochemical performances, demonstrating their potential applications in flexible smart windows that combine energy storage and electrochromism. 

### 4.2. Electroluminescent Device 

OLEDs are research hotspots in recent years due to their advantages of fast reaction time, low operating voltage, high contrast, large size and flexible panels. At present, OLEDs are widely used in display panels of mobile phones (small screens) and televisions (large screens). Moreover, by comparing the OLED’s top emission and the bottom emission, it is found that the top emission device has better energy saving and longer service life. Therefore, most of the studied OLEDs currently are top emitting devices, and in the topemitting devices the selection of transparent electrodes is the most important factor. A suitable transparent electrode will greatly improve the light transmittance and conductivity of the device. Ag NWs TCE is widely used in OLEDs because of its excellent conductivity and transparency, in addition, the Ag NWs TCE has good flexibility compared with the widely used ITO TCE, and can be used as electrode for flexible OLEDs. 

Lian et al. [104] prepared a highly conductive, low-roughness transparent electrode composed of alginate/Ag NWs by ambient temperature solution method. The assembled diode has a current density and brightness comparable with that of an ITO anode, and it has a good charge balance, high efficiency, and a current efficiency of 1.4 times that of ITO. Alshammari et al. [117] used TN/PEDOT:PSS composite to fabricate TCE for OLED by combining nanocomposite and solution-based methods. The TCE prepared in this study meets the requirements of practical applications with a maximum brightness of 7 × 10^3^ cd/m^2^, similar as ITO-based devices, can be used as an excellent alternative to ITO. Kim et al. [118] developed a hybrid film of PEDOT:PSS/Ag NWs, which can be used as a transparent electrode in a flexible electrochemical luminescence (ECL) display. The reportedresults showed that PEDOT:PSS/Ag NWs film with non-ionic surfactant is fabricated. There is excellent electrical stability and light transmittance when it is severely bent (bending radius = 5 mm). Moreover, the ECL display prepared by this electrode exhibited high operational stability even after 1000 cycles of bending test, as shown in Figure 6c. It is indicated that the hybrid electrode has broad application prospects in the development of flexible displays. Lee et al. [119] enhanced the outcoupling of OLED devices by introducing microporous polyimide films on the backside of Ag NWs TCE embedded in pure colorless polyimide (PI), and the preparation of the conductive and scattering flexible substrates is schematically illustrated in Figure 6b. This strong external coupling OLED not only has high thermal stability (>360 °C), chemical and mechanical stability, but also could improve the color uniformity of the viewing angle, which is an important feature in lighting applications. Therefore, the composite of Ag NWs/scattering PI substrate provides a flexible platform for future lighting applicationsin efficient white OLED applications. 

### 4.3. Thin-Film Heater 

Hyperthermia is a popular physical therapy that is very effective in treating joint pain and fatigue, but conventional medical hyperthermia instruments are rigid and non-portable, and are becoming more flexible as there is more interests in wearable electronics. Flexible film devices that are closely attached to the skin or clothing have attracted widespread attentions, promoting the development of stretchable and transparent heated films. The traditional ITO is brittle, which limits the development of TFHs based on ITO TCEs. Ag NWs are excellent in electrical conductivity and thermal conductivity, gaining attentions in the preparation of TFHs and are rapidly developed.

Cheong et al. [120] studied a highly flexible transparent TFH (f-TFH) consisting of Ag NWs and aluminum zinc oxide (AZO). It is found that the Ag NWs TCE formed after coating AZO weakened the heat convection in the air and the thermal efficiency of the heater is improved, and a higher average film temperature (T_ave_) is obtained by adjusting the ratio of the area coverage of Ag NWs to AZO, which achieves uniformity at higher temperature over the entire plane. In the same year, Cheong et al. [121] reported a mixture of Ag-based NWs networks and metals or metal oxides formed by spin coating, sputtering, etc. on flexible substrate of PI. In this study, Ag NPs and ITO are sputtered on the basis of Ag NWs, and the mixed film is found to have lower sheet resistance (Rs) and enhanced mechanical reliability, and to be higher in mixed TFH. In addition, the TFH showed highly uniform heat distribution over the areas.

Jang et al. [122] prepared a flexible stretchable film heater based on Ag NFs TCE by electrospinning. It is found that the area fraction of Ag NFs network can be adjusted by duration of the electrospinning process to control the photoelectric performances of the film. This adjusts the temperature range and power consumption of the heater. Moreover, due to the high flexibility, stretchability and thermal conductivity of Ag NFs TCE, the heater can be closely attached to human skin and can maintain a uniform temperature distribution under mechanical deformation. The temperature profile is uniform and the heating and cooling rates are faster than those of ITO. Singh et al. [123] prepared an embedded PVA/Ag NFs network by electrospinning for ultra-smooth, high-performance TCEs. Based on this, a flexible TFH is developed with excellent performance.

Lan et al. [15] prepared a flexible transparent TFH by embedding Ag NWs into a PVA film (Ag NWs/PVA). The Ag NWs/PVA film is found to have excellent optical properties, electrical conductivity, environmental endurance. It has good mechanical flexibility under various harsh conditions such as compression and heating. It overcomes the lack of flexibility of traditional TFHs in use. The TFH can fully contact with the skin and even the joints. Further integration with flexible substrates has great potential in medical devices such as thermal pads.

### 4.4. Electrochromic Film

Electrochromism refers to the process of stable, reversible change of transmittance, reflectance or absorptivity of UV, visible or near-infrared regions under the action of an applied electric field. It is intuitively expressed as the color of the material and the phenomenon of reversible change in transparency has the characteristics of high efficiency, low consumption, green and intelligent technology. Traditionally, ITO films have important applications in EC devices as TCEs. However, according to the disadvantages of ITO mentioned above, Ag NWs TCE with high transparency, good electrical conductivity and mechanical flexibility has gained wide attentions as an alternative to ITO TCE.

Among them, EC smart windows are considered as the most promising alternatives to traditional dimming equipment such as curtains and glass stickers. Lin et al. [124] developed a roller-roller preparation method that does not require heat treatment. Mass production of flexible, ultra-large, transparent Ag NFs network electrodes is achieved, and the assembly of prepared TCEs into A4 size EC smart windows (ECSWs) found that these ECSWs have short switching time, high coloring efficiency, and good flexibility. The advantages and higher performance than ITO-based commercial devices provide the possibility for Ag NFs TCEs in large flexible electronic devices such as ECSWs and curved displays that are flexible and deformable. The fabrication and properties of Ag NFs ECSW are shown in Figure 6d. 

Kim et al. [125] prepared a high transmittance and conductivity TCE based on Ag NWs/PEDOT:PSS by spraying. In this study, the performance of the electrode is optimized by adjusting the ink formulation. The results showed that this hybrid electrode has a lower sheet resistance than those of ITO TCE and Ag NWs TCE. Moreover, when we use the hybrid electrode for EC devices, it has a very different optical response and high coloring efficiency, and has very good application value. 

### 4.5. Solar Cells 

As the energy crisis and environmental pollution problems become more serious, the development of new clean energy sources is extremely important. Solar energy is the most promising new clean energy technology due to its environmental protection, high efficiency and inexhaustible advantages. In recent years, solar cells are developed rapidly. Transparent conductive oxide electrode is generally used as the photoelectric electrode of the solar cell. The most commonly used material is ITO. However, ITO has high manufacturing cost, a complicated manufacturing process, toxicity, poor bending resistance, high temperature heating, etc. Ag NWs with potential applicationshave their own advantages in the preparation of solar cells due to their excellent performance. Alami et al. [92] used a Ag NWs grid as the photoelectrode of a dye-sensitized solar cell, which has the advantage of high-spectrum transmission in the visible–near-infrared region. It also offers greater flexibility and high electrical conductivity compared with conventional ITO based optoelectronic electrodes. 

At present, perovskite solar cells (PSCs) are gained wide attentions due to their advantages of high efficiency, low cost, large-area production and the preparation of translucent cells. Zhang et al. [126] reported an ITO/Ag-grid/AZO hybrid electrode for a planar perovskite solar cell fabricated at a temperature of 150 °C. This electrode exhibited very low sheet resistance, high transparency and a power conversion efficiency of 13.8%, indicating that the use of ITO/Ag-grid/AZO hybrid electrode in the manufacture of perovskite solar cells will improve power conversion efficiency (PCE) and reduce the cost.

Double-sided translucent PSCs have great potential for improving the efficiency of tandem solar cells and expanding double-sided photovoltaic applications. Pang et al. [127] reported an effective three-layer structure, polyethylene amine ethoxylate/Ag/molybdenum oxide (PEIE/Ag/MoO_x_), as a cathode of solar cell, increasing the device PCE to 10.40% (ITO side) and 6.54% (Ag side). This solar cellshowed better double-sided performance by optimizing the thickness of Ag and introducing a high refractive index MoOx optical coupling layer outside the Ag electrode.

Ag-grid electrodes due to their high electrical conductivity and high optical transparency have become candidate electrodes for use as TCEs in large-area flexible thin film photovoltaic cells. However, in PSCs, the corrosion of the Ag electrode by perovskite greatly inhibits the performance and stability of the device. For this problem, Wang et al. [128] introduced the ammonia: polyethyleneimine modified PH1000 (highly conductive PEDOT:PSS) to suppress this corrosion, and a high-performance flexible PSC with 14.52% PCE is prepared on a PET/Ag-grid/modified high conductivity PEDOT:PSS (PH1000) flexible composite transparent electrode. The experimental results showed that the flexible PSC using the composite electrode exhibited excellent robustness and durability, and maintained 86% of the initial performance after 5000 full bending cycles. 

### 4.6. Stretchable Devices 

With the rapid development of wearable devices and the increase of people’s demand, stretchable electronics are attracting significant attentions compared with the traditional rigid electronic devices, due to their flexibility and stretchability [129,130]. This kind of devices can not only bend, but also adapt to large deformation, so they have a broad application prospect. Due to the excellent properties of Ag NWs, stretchable devices based on Ag NWs conductors have been extensively studied. 

On the one hand, Ag NWs are embedded into the polymer substrate. Jun et al. [131] combined Ag NWs with a thermoplastic polyurethane (TPU) surface by intense pulsed light irradiation to construct the embedded structure of Ag NWs/TPU. Surface fusion ensures that the patterned Ag NWs adhere to the TPU even when stretched at a strain greater than 33%, and this embedded structure showed high tensile performance. Qiang et al. [132] embedded vein-Ag NWs into polydimethylsiloxane (PDMS) by a simple impregnation process. The vein-Ag NWs-PDMS conductor can maintain high conductivity after 150% mechanical elongation. Due to its excellent performance and simple manufacturing process, the vein-Ag NWs-PDMS conductor has great potential in stretchable electronic devices. In the stretchable electronic devices with Ag NWs as functional materials, the binding force between Ag NWs and the high-elastic substrate has a great influence on the tensile performance of the devices. In order to enhance the stretchability of the device, You et al. [133] made many attempts, including simply depositing Ag NWs on the surface of PDMS or preparing a sandwich structure of PDMS-Ag NWs-PDMS, and found that Ag NWs are easy to detach from the PDMS after repeated stretching and bending. To solve this problem, You et al. used polyurethane urea (PUU), a stretchable and transparent polymer, which is compatible with Ag NWs and PDMS in the experiment. Strong hydrogen bonding between the three enhances the stability. The device is tested to stretch to 150% and passed 5000 cycles of 100% stretch–release testing. 

On the other hand, Ag NWs are embedded into ink and printed to make Ag NWs-based conductors. With the development of printing electronic technology, a growing number of researchers are investigated the devices based on patterned Ag NWs network, such as Cai et al. [134] printed Ag NWs inks with a length of about 40 μm to various substrates by direct writing and they found that the longer NWs tend to have greater curvature and better ability to maintain good line-to-line contact stretching. Huang et al. [135] printed high-concentration Ag NWs inks on a flexible substrate via inkjet printing, then spin-coated liquid PDMS on printed Ag NWs graphics to form stretchable conductors and demonstrated that stretchable heaters could be prepared. Except direct printing and inkjet printing, EHD printing technology is also used in the Ag NWs printing process. Cui et al. [21] used EHD printing technology to develop Ag NWs ink for printing on various substrates, and performed bending and stretching characterizations of Ag NWs samples on PDMS, and then demonstrated the potential of EHD printing technology in Ag NWs-based flexible and stretchable equipment by fabricating the Ag NWs heater and electrocardiogram electrode. With the development of Ag NWs-based high-performance conductors, stretchable devices have broad application prospects.

## 5. Conclusions

As an important kind of material with high conductivity and light transmittance, Ag NWs are unique candidate for transparent electrodes applied in microdevices/systems. This review mainly introduces the synthesis of Ag NWs, the preparation of Ag NWs TCE, and their applications in flexible energy storage devices, electroluminescent devices, EC devices, TFHs and solar cells. With the introduction of printing technology and the development of synthesis of Ag NWs, Ag NWs-based electroluminescent devices and solar cells are in rapid development. However, the practical applications of Ag NWs TCE still face some critical challenges, such as the reservation of morphology in the synthesis process, the preparation process of TCE with high node resistance, mechanical stability, thermal stability, environmental stability, and the realization of stability and feasibility of mass production, etc. 

## Figures and Tables

**Table 1:** Comparison of properties of several TCEs.

Performance	ITO	Ag NWs	CNT	Graphene	Ag Nano Network
Sheet resistance	—	—	++	++	——
Transmittance	++	+	+++	++	+++
Stability	++	+++	++	++	+++
Flexibility	——	+++	+++	+++	—
Large scale	——	++	++	++	——
Fabricating cost	+++	—	++	+++	+++
